# Identification of miRNAs that target Fcγ receptor-mediated phagocytosis during macrophage activation syndrome

**DOI:** 10.3389/fimmu.2024.1355315

**Published:** 2024-03-15

**Authors:** Kontham Kulangara Varsha, Xiaoming Yang, Alkeiver S. Cannon, Yin Zhong, Mitzi Nagarkatti, Prakash Nagarkatti

**Affiliations:** Department of Pathology, Microbiology and Immunology, School of Medicine, University of South Carolina School of Medicine, Columbia, SC, United States

**Keywords:** cytokine, Fcγ receptors, MAS, MiRNA mimics, MiRNA inhibitors, miRNA therapeutics, phagocytosis

## Abstract

Macrophage activation syndrome (MAS) is a life-threatening complication of systemic juvenile arthritis, accompanied by cytokine storm and hemophagocytosis. In addition, COVID-19–related hyperinflammation shares clinical features of MAS. Mechanisms that activate macrophages in MAS remain unclear. Here, we identify the role of miRNA in increased phagocytosis and interleukin-12 (IL-12) production by macrophages in a murine model of MAS. MAS significantly increased F4/80+ macrophages and phagocytosis in the mouse liver. Gene expression profile revealed the induction of Fcγ receptor–mediated phagocytosis (FGRP) and IL-12 production in the liver. Phagocytosis pathways such as High-affinity IgE receptor is known as Fc epsilon RI -signaling and pattern recognition receptors involved in the recognition of bacteria and viruses and phagosome formation were also significantly upregulated. In MAS, miR-136-5p and miR-501-3p targeted and caused increased expression of Fcgr3, Fcgr4, and Fcgr1 genes in FGRP pathway and consequent increase in phagocytosis by macrophages, whereas miR-129-1-3p and miR-150-3p targeted and induced Il-12. Transcriptome analysis of patients with MAS revealed the upregulation of FGRP and FCGR gene expression. A target analysis of gene expression data from a patient with MAS discovered that miR-136-5p targets *FCGR2A* and *FCGR3A/3B*, the human orthologs of mouse Fcgr3 and Fcgr4, and miR-501-3p targets *FCGR1A*, the human ortholog of mouse Fcgr1. Together, we demonstrate the novel role of miRNAs during MAS pathogenesis, thereby suggesting miRNA mimic–based therapy to control the hyperactivation of macrophages in patients with MAS as well as use overexpression of FCGR genes as a marker for MAS classification.

## 1 Introduction

Macrophage activation syndrome (MAS) is a life-threatening inflammatory condition that occurs as a result of the uncontrolled and defective immune reaction following the expansion and activation of T cells and macrophages, leading to cytokine storm and hemophagocytosis (1, 2). Hemophagocytosis is characterized by phagocytosis of erythrocytes, lymphocytes, or other hematopoietic precursors by macrophages. Clinical characteristics of MAS are defined to be high fever, hepatosplenomegaly, and central nervous system disorders. Laboratory identification features include peripheral pancytopenia, hyperferritinemia, elevated liver enzymes, and reduced fibrinogen and activated macrophages with phagocytic activity (1, 3).

MAS constitutes a fatal complication associated with certain systemic inflammatory diseases such as hemophagocytic lymphohistiocytosis (HLH), systemic juvenile idiopathic arthritis (sJIA), and adult-onset Still’s disease. Moreover, the occurrence of MAS is also reported among patients with Kawasaki disease, systemic lupus erythematosus, patients with cancer receiving immunotherapy, and, most recently, MAS-like condition has been reported among patients with COVID-19 (4–7). Regardless of the association of MAS with various disease conditions, little is known about the etiology and pathogenesis of this disorder. Multiple recent publications tried to establish the role of pro-inflammatory cytokines, such as interleukin-18 (IL-18), interferon-γ (IFN-γ), and IL-1β on MAS pathogenesis and proposed the detection of these cytokines as biomarkers to classify MAS as well as cytokine antagonists to treat the disease (8–11).

While increased cytokine production and phagocytosis by macrophages are considered to be the characteristic features of MAS, the precise mechanisms that lead to such macrophage activation remain unclear. miRNAs are short (20–24 nucleotides in length) single-stranded RNA molecules that can post-transcriptionally regulate the expression of hundreds of transcripts in the same cells (12). It is estimated that more than 60% of human coding transcripts are regulated by miRNAs (13). miRNAs have been shown to regulate macrophage activation and differentiation, including polarization from a pro-inflammatory M1 state to an anti-inflammatory M2 phenotype (12). Whether miRNA regulates the functions of macrophages during MAS has not been previously investigated. In the current study, therefore, we investigated the miRNA alterations occurring during MAS using a well-characterized mouse model, in which unmethylated cytosine–guanine dinucleotide (CpG), a Toll-like receptor 9 (TLR-9) ligand, is repeatedly injected into C57BL/6 mice to induce MAS (14).

Specifically, we tried to identify miRNAs that regulate increased Fcγ receptor (FCGR)–mediated phagocytosis (FGRP) and IL-12 production by macrophages during MAS. Using miRNA target analysis and transfection studies, we identified unique miRNAs that targeted genes in FGRP pathway and promoted phagocytosis as well as IL-12 production. Our studies shed new light on the role of miRNA in regulating the excessive activation and functions of macrophages in MAS.

## 2 Material and methods

### 2.1 Mice and CpG treatment

Female C57BL/6 mice, aged 10–12 weeks, were purchased from Jackson Laboratories (Bar Harbor, USA). Mice were given access to water and standard diet *ad libitum* and maintained in our Association for Assessment and Accreditation of Laboratory Animal Care–certified animal facility under controlled temperature and humidity and pathogen-free conditions with 12-h dark/12-h light cycles. Mice were injected intra peritoneal with a total of five doses (50 µg) of CpG (InvivoGen) prepared in phosphate-buffered saline (PBS) as reported before (14). Control mice were injected with PBS alone. A complete blood count was tested on the eighth day using the VetScan Hematology analyzer (Zoetis, USA). Mice were euthanized on the 10th day, and organs and serum were collected for analysis.

#### 2.1.1 Study approval

All experiments were performed according to the protocol, AUP-2669-101803 approved by the University of South Carolina Institutional Animal Care and Use Committee.

### 2.2 ELISA

Mouse tumor necrosis factor–α (TNF-α), IL-12/23, IL-12, IL-6, and IFN-γ enzyme-linked immunosorbent assay (ELISA) kits were purchased from BioLegend, USA. Mouse Ferritin ELISA kit was procured from ALPCO Diagnostics, USA. All kits were used according to the instructions of manufacturers, as detailed in our previous work (15).

### 2.3 Isolation of liver MNCs

The liver was collected 24 h after the administration of the last dose of CpG, and liver mononuclear cells (MNCs) were isolated using a liver dissociation kit (Miltenyi Biotec, Germany) according to the protocol published elsewhere by our laboratory (16). In short, the whole liver inside the MACS C tube with the enzyme mix was homogenized using a gentleMACS™ dissociator and filtered according to the manufacturer’s instructions. The cell suspension was washed with 5 mL of Dulbecco’s modified Eagle medium (DMEM) at 300×g for 10 min, and the cell pellet was resuspended in 6 mL of staining buffer [PBS containing 2% heat-inactivated fetal bovine serum (FBS) and 1 mM ethylenediamine tetraacetic acid (EDTA)] and transferred to 3 mL of 100% Percoll. The cell pellet was collected following centrifugation at 2,000 rpm for 15 min, and red blood cell lysis (RBC lysis buffer, Roche) was performed for 5 min on ice. The cells were filtered using a 70-µm filter and washed with staining buffer. The resulting single cells were used for analyses.

### 2.4 Flow cytometry analysis

Liver MNCs were counted using TC20 Automated Cell Counter (Bio-Rad, USA), and 1 × 10^6^ cells were used for analysis as reported before (17). Cell Fc receptors were blocked by 10-min incubation with TruStain FcX (BioLegend) following which the cells were incubated with fluorochrome-conjugated antibodies for 30 min. Antibodies used are CD45, CD11B, F4/80, LY6C, LY6G, and CD11C. All antibodies were purchased from BioLegend, USA. All incubations were performed on ice, and the cells were washed with the staining buffer and analyzed by BD FACS Celesta flow cytometer. Data analysis was performed by FlowJo v10 software.

### 2.5 RNA sequencing

RNA was isolated from liver MNCs using the RNeasy Minikit (Qiagen, Hilden, Germany) and quantified by the Qubit High Sensitivity RNA Assay Kit (Thermo Fisher, Waltham, MA). A NEBNext Ultra II RNA library prep kit for Illumina (New England Biolabs) was used to prepare the RNA library following the instructions of the manufacturer. Sequencing of the libraries was performed on a NextSeq 550 Sequencer (Illumina). Data analysis was done using Qiagen’s RNA analysis portal using default settings with strand specificity set to reverse. Pathway enrichment analysis was performed by ingenuity pathway analysis (IPA) software (Qiagen) as described (18).

### 2.6 miRNA sequencing

Total RNA was isolated from liver MNCs using the MiRNeasy Minikit (Qiagen, Hilden, Germany) and quantified by the Qubit High Sensitivity RNA Assay Kit (Thermo Fisher, Waltham, MA). Total RNA from five mice of each group was pooled together, and miRNA libraries were prepared using the QIAseq miRNA Library Kit (Qiagen) according to the manufacturer’s instructions. Sequencing of the libraries was performed on a NextSeq 550 Sequencer (Illumina) as described previously (18). Data analysis was done using Qiagen’s RNA analysis portal using default settings. Differential expression analysis was performed using the same platform with default settings. miRNA target identification was performed by IPA, and functional enrichment analysis was performed by GeneCodis (19).

### 2.7 qRT-PCR

For miRNA validation, cDNA was synthesized from liver MNCs using the miRCURY locked nucleic acids (LNA) RT Kit (Qiagen), and quantitative real-time PCR (qRT-PCR) was performed by a miRCURY LNA SYBR Green PCR kit (Qiagen), as described (20). All the LNA miRNA primers were purchased from Qiagen, and U6 snRNA (v2) was used as internal control. Data analysis was performed by the comparative Ct (ddCt) method (21).

For validation of genes, cDNA was synthesized from liver MNCs using the iScript cDNA synthesis kit (Bio-Rad), and qRT-PCR was performed using SsoAdvanced Universal SYBR Green Supermix (Bio-Rad). Forward and reverse primers were purchased from IDT (Integrated DNA Technologies, USA) and 18S, or Gapdh was used as an internal control. Data analysis was performed by the ddCt method. Primers used for validation of miRNAs, and genes are provided in **Supplementary Tables 2, 3**.

### 2.8 Transfection for gene expression analysis

Transfections of macrophages isolated from mice were performed using miRCURY LNA miRNA mimics and miRCURY LNA miRNA power inhibitors (Qiagen), as described (18). For control, negative control of mimic and negative control of power inhibitor were used. Transfections were performed using HiPerfect transfection reagent (Qiagen). Briefly, liver MNCs were isolated from MAS mice as described above, and macrophages were isolated from liver MNCs using the EasySep™ Mouse F4/80 Positive Selection Kit (STEMCELL Technologies) following the protocol provided by the manufacturer. Two million macrophages were transfected with 50 nM concentration of mimic, 50 nM concentration of inhibitor, and respective control independently and incubated at 37°C and 5% CO2 for 4 days in DMEM containing heat-inactivated 10% FBS and 1% penicillin/streptomycin.

### 2.9 Transfection for phagocytosis assay

For the phagocytosis assay, 1 × 10^5^ RAW 264.7 macrophage cells were seeded and after 24 h transfected with 50 and 70 nM inhibitors of miR-136-5p, miR-501-3p (70 nM alone for Immunoglobulin G (IgG) phagocytosis assay), and respective control as explained above. The culture medium was replaced with fresh medium after 24 h and incubated for another 24 h. Double transfection was performed for phagocytosis assay using mimics. Briefly, 1 × 105 RAW 264.7 cells were transfected with 50 nM mimics of miR-136-5p, miR-501-3p, and respective control as explained above, and the medium was replaced with fresh culture medium after 24 h of incubation. After 24 h, the medium was removed, and the cells were transfected with 25 nM respective mimics and control. Culture medium was replaced after 24 h and incubated for another 24 h.

### 2.10 Phagocytosis assay

#### 2.10.1 IgG phagocytosis assay

To quantitate Fcgr-mediated phagocytosis, following transfection of 1 × 10^5^ RAW 264.7 cells with mimics and inhibitors, Cayman’s IgG (fluorescein isothiocyanate (FITC)-conjugated) phagocytosis kit (Cayman, 500290) was used according to the manufacturer protocol. Briefly, following incubation in a 96-well black polystyrene clear-bottom tissue culture–treated microplate (Corning), the culture medium was removed, and IgG particles prepared in DMEM were added to the wells. Following incubation of 1 h at 37°C, the medium with IgG was removed, cells were washed with PBS, and cell surface–bound fluorescent particles were quenched by trypan blue. After PBS wash, fluorescence was measured at emission excitation of 465/540 and represented as total radiant efficiency. For imaging, IgG particles were removed and washed with PBS. Cells were stained with NucBlue live-ready probes (Hoechst 33342 dye) reagent (Invitrogen). The stain was removed after 10 min of incubation and washed with PBS. Live cell imaging solution was added to the cells and imaged using Evos FL Auto 2 (Invitrogen).

#### 2.10.2 pHrodo deep red E. coli bioparticles phagocytosis assay

To quantitate phagocytosis following transfection of 1 × 10^5^ RAW 264.7 cells with mimics and inhibitors, pHrodo deep red *E. coli* bioparticles conjugate for phagocytosis (Invitrogen, P35360) were used according to the manufacturer’s protocol. Briefly, following incubation in a 96-well black polystyrene clear-bottom tissue culture–treated microplate (Corning), the culture medium was removed, and bioparticles prepared in live cell imaging solution (Invitrogen) were added to the wells. Following incubation of 45 min at 37°C, fluorescence was measured at emission excitation of 640/680 and represented as total radiant efficiency. For imaging, the bioparticles were removed and washed with PBS. Cells were stained with NucBlue live-ready probes (Hoechst 33342 dye) reagent (Invitrogen). The stain was removed after 10 min of incubation and washed with PBS. Live cell imaging solution was added to the cells and imaged using Evos FL Auto 2 (Invitrogen).

### 2.11 *In vivo* phagocytosis assay

pHrodo deep red *E. coli* bioparticles conjugate for phagocytosis were used to quantify phagocytosis *in vivo* according to a previous report (22). For this, we generated MAS in mice as described before, and, 24 h after the last dose, 1 mg of bioparticles was administered intravenously. PBS administered mice were used as control. After 2 h, the mice were euthanized, the liver was collected, and fluorescence was quantified and imaged using IVIS Spectrum *in vivo* imaging system (PerkinElmer) with emission excitation set as 640/680.

### 2.12 Studies in patients with MAS

The whole RNA sequencing raw data and microarray data of four patients with MAS and normal controls were retrieved from Gene Expression Omnibus (GEO). Sequencing data were analyzed using CLC-Genomics workbench software (Qiagen) with default settings. Microarray data were analyzed by Transcriptome Analysis Console software (Thermo Fisher). IPA was used for pathway analysis.

### 2.13 Statistics

Statistical analyses were performed by Prism (GraphPad software). Unpaired Student’s t-test was used for comparisons, and data were represented as mean ± SEM. Data were considered significant when *p* is <0.05.

## 3 Results

### 3.1 Immune profile in CpG-induced MAS

**Figure 1:**
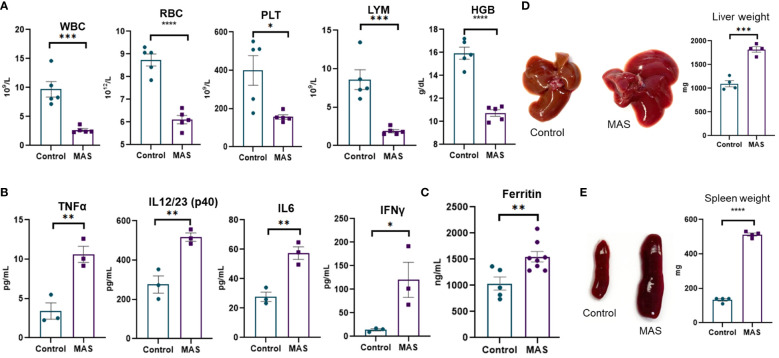
Mice with MAS display peripheral pancytopenia, cytokine storm and hepatosplenomegaly. Mice were given CpG as described in Materials and methods to induce MAS, and, on day 10, the mice were euthanized for the analysis as follows. **(A)** Complete blood count analyses of control and CpG treated mice (n = 5). **(B)** Measurement of inflammatory cytokines using ELISA assays of blood serum collected from control and mice with MAS (n = 3). **(C)** Measuring ferritin in the serum using ELISA (n = 8). **(D)** Measurement of liver weight (n = 4). **(E)** Measurement of spleen weight (n = 4). Vertical bars represent mean ± SEM. MAS and control mice were compared for statistical significance using Student’s t-test. *p < 0.05, **p < 0.01, ***p < 0.005, and ****p < 0.0001.

Multiple injections of the TLR-9 agonist, CpG, has been previously shown to induce MAS-like disease in the mouse (14). In the current study, we investigated the immune profile of such mice. We noted peripheral pancytopenia with a significant decrease in white blood cells (WBCs), red blood cells (RBCs), platelets, lymphocytes, and hemoglobin in MAS-bearing mice (**Figure 1A**) when compared to the vehicle controls. Cytokine storm with elevated inflammatory cytokines was observed in MAS mice, with significant increases in TNF-α, IL-12/23, IL-6, and IFN-γ in the serum (**Figure 1B**). Elevated ferritin, an important clinical characteristic of MAS was also found to be increased in the serum of MAS mice when compared to the control mice (**Figure 1C**). Hepatomegaly and splenomegaly, the other clinical features identified in patients with MAS, were well distinguished in the disease group with marked increase in the size and weight of the liver and spleen when compared to the controls (**Figures 1D, E**).

### 3.2 MAS triggers macrophages, monocytes, and dendritic cells

**Figure 2:**
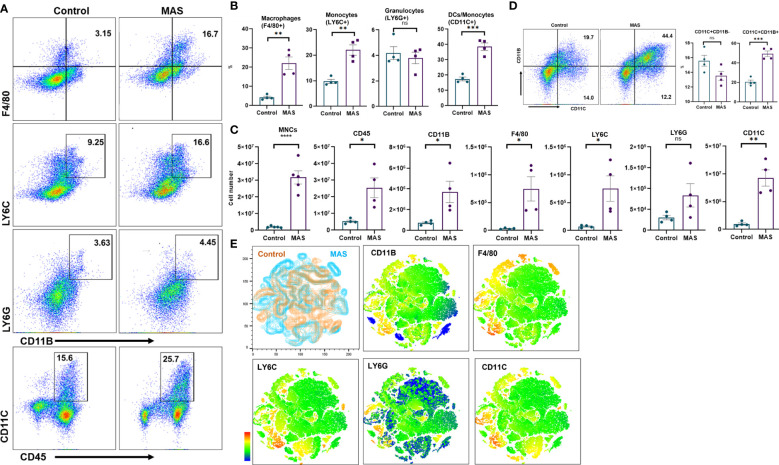
An increase in macrophage and monocyte populations is observed in the liver MNCs of mice with MAS. Liver MNCs were isolated from control (n = 4) and MAS mice (n = 4) and subjected to flow cytometry analysis. **(A)** Flow plots showing staining of liver MNCs with respective antibodies. **(B)** Percentage of F4/80+ cells representing macrophages, LY6C+ cells indicating monocytes, and CD11C+ cells representing DCs/monocytes. **(C)** Total number of respective cell type. **(D)** Flow plots and percentage of CD11C and CD11B population. **(E)** Control (brown) and MAS (blue) mice t-SNE plots generated for the markers CD11B, F4/80, LY6C, LY6G, and CD11C. Vertical bars represent mean ± SEM. MAS and control mice were compared for statistical significance using Student’s t-test. *p < 0.05, **p < 0.01, ***p < 0.005, and ****p < 0.0001.

Uncontrolled activation of macrophages has been reported in patients with MAS (23). However, an account of the status of macrophages and other immune cells is not methodically investigated in preclinical models. Here, we examined the percentage and total number of myeloid cells in the liver of mice during the occurrence of MAS by flow cytometry. Representative flow cytometric analysis with the percentages of myeloid cells has been shown in **Figure 2A**. These data demonstrated that MAS caused a significant increase in the percentage of macrophages, monocytes and dendritic cells (DCs) in MAS-bearing mice with no significant change in granulocytes, when compared to control mice (**Figure 2B**). When we analyzed the total number of myeloid cells in the liver, MAS-bearing mice showed a substantial increase in the number of MNCs in the liver when compared to the vehicle-treated control mice (**Figure 2C**). Significant increase in the number of CD45+ CD11B+ cells indicated an upsurge of cells of myeloid lineage in MAS mice. Further explorations revealed a significant increase in the absolute number of F4/80+ macrophages, LY6C+ monocytes, and CD11C+ DCs/monocytes (**Figure 2C**). However, no significant variation was observed in LY6G+ granulocytes (**Figures 2B, C**), which is in line with a previous report (14). Interestingly, there was no increase in the percentages of CD11C+CD11B− DCs, whereas a remarkable increase in the proportions of CD11C+CD11B+ cells were observed (**Figure 2D**). A subset of DCs, monocytes, and macrophages was reported to express CD11C and CD11B together (24). The t-distributed Stochastic Neighbor Embedding (t-SNE) plots of each antibody staining generated for both control and MAS mice are illustrated in **Figure 2E**. Control and MAS liver MNC data were concatenated to qualitatively visualize and compare each antibody staining. A continuous color scale from blue to red indicated the intensity of expression of each marker with blue being the lowest. The t-SNE maps showed increased staining of CD11B, F4/80, LY6C, and CD11C from MAS liver MNCs.

### 3.3 MAS alters the expression of genes and miRNAs involved in phagocytosis and IL-12 production

**Figure 3:**
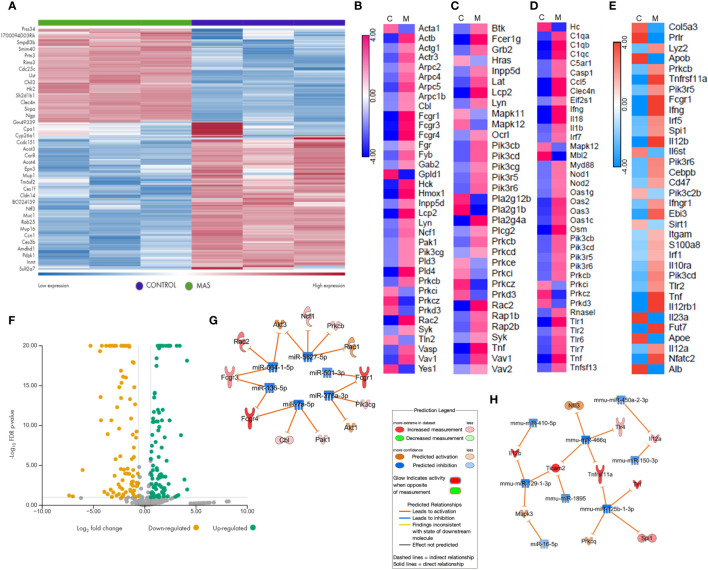
Gene and miRNA expression profiling of liver MNCs following miRNA and RNA sequencing. **(A)** Gene expression profile of top upregulated and downregulated genes in control and mice with MAS. Heat map showing expression pattern of genes involved in **(B)** Fcγ receptor–mediated phagocytosis, **(C)** Fcϵ RI signaling, **(D)** role of pattern recognition receptors in recognition of bacteria and viruses, and **(E)** Il-12 production by macrophages and monocytes. **(F)** Volcano plot of significantly altered miRNAs in MAS-bearing mice. Target predictions of miRNAs regulating **(G)** Fcγ receptor–mediated phagocytosis and **(H)** Il-12 production by macrophages.

We performed RNA sequencing of the liver MNCs of three mice from each group. The heat map generated following gene expression analysis demonstrated a strong correlation in gene expression among all three mice from the same group (**Figure 3A**), and there were significant differences in the gene expression profiles of MAS vs control groups (**Figure 3A**). Differential expression and pathway enrichment analyses revealed elevated expression of genes involved in various phagocytosis pathways such as FGRP in macrophages and monocytes (**Figure 3B**), Fc epsilon RI (FcϵRI) signaling (**Figure 3C**), pattern recognition receptors (PRRs) in recognition of bacteria and viruses (**Figure 3D**), as well as IL-12 signaling and production in macrophages (**Figure 3E**). Remarkably, a high expression of the upstream receptor genes of the FGRP pathway such as Fcgr1 (6.28-fold change), Fcgr3 (3.8-fold change), and Fcgr4 (13.8-fold change) was noticed (**Figure 3B**), which suggested an increase in phagocytosis by macrophages during MAS. Additionally, a marked increase in the expression of Il12a, encoding the p35 sub-unit of Il12 (2.3-fold change) and Il12b, encoding the p40 sub-unit of Il12 (6.4-fold change) genes was observed (**Figure 3E**). Next, we investigated if MAS induction altered the expression of miRNA. miRNA sequencing of liver MNCs revealed an altered expression of 513 miRNAs (1.5-fold change) in MAS mice when compared to the controls. Significantly altered miRNAs are shown in **Figure 3F**. Target analysis of the miRNA data set with concatenated gene expression data identified miRNAs involved in the canonical pathways FGRP in macrophages and monocytes as well as IL-12 signaling and production in macrophages (**Figures 3G, H**).

### 3.4 miRNAs altered during MAS are associated with central nervous system disorders

Functional enrichment analysis of significantly altered miRNAs by GeneCodis identified sensorineural hearing loss as one major disease that is reported to be associated with chronic inflammatory conditions such as rheumatoid arthritis (25, 26) along with long term synaptic depression (**Supplementary Figure 1A**), which is the reduced efficacy of synaptic transmission of neuronal signals (27). IPA identified neurological diseases and psychological disorders (**Supplementary Figure 1B**), which are clinical characteristics of MAS as diseases associated with differential expression of miRNAs in MAS. Kyoto Encyclopedia of Genes and Genomes (KEGG) pathway analysis by GeneCodis identified pathways related to central nervous system disorders (**Supplementary Figure 2A**), whereas neuron, dendrite, and postsynaptic membrane were identified as the cellular components associated with the miRNAs regulated during MAS (**Supplementary Figure 2B**).

### 3.5 miRNAs modulate the expression of upstream receptors of FGRP and subunits of Il-12 in mouse macrophages

**Figure 4:**
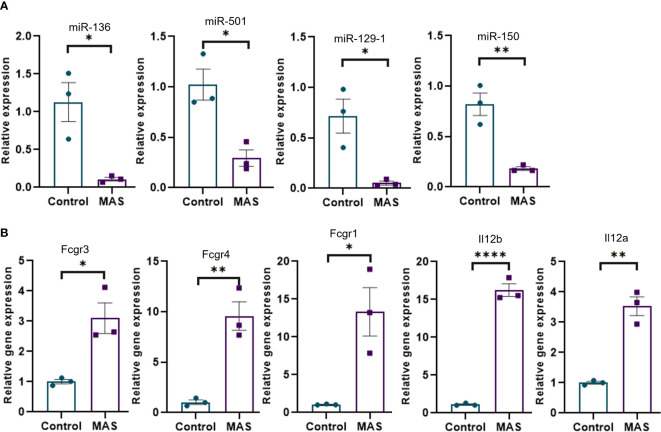
Validation of selected miRNAs and their targets by qRT-PCR. **(A)** Relative expression of miRNAs modulating Fcγ receptor–mediated phagocytosis and Il-12 production by macrophages and monocytes. **(B)** Relative expression of targets of miR-136 (Fcgr3 and Fcgr4) and miR-501 (Fcgr1) involved in Fcγ receptor–mediated phagocytosis and targets of miR-129-1 (Il12b) and miR-150 (Il12a) involved in Il-12 production. Vertical bars represent mean ± SEM. MAS and control mice were compared for statistical significance using Student’s t-test. *p < 0.05, **p < 0.01, and ****p < 0.0001.

Concatenation of miRNA-seq data with mouse RNA-seq data identified miR-136-5p to be targeting Fcgr3 and Fcgr4, miR-501-3p targeting Fcgr1, miR-129-1-3p targeting Il12b, and miR-150-3p targeting Il12a. Next, we performed the validation of miRNAs and mRNAs by qRT-PCR. The data showed that miR-136-5p, miR-501-3p, miR-129-1-3p, and miR-150-3p were downregulated, whereas their respective target genes were upregulated in the liver MNCs of MAS mice when compared to the controls (**Figures 4A, B**).

**Figure 5:**
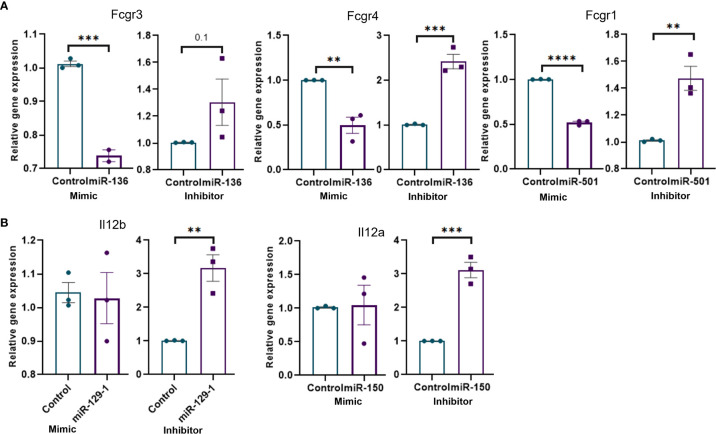
Transfection of mouse macrophages using miRNA mimics and inhibitors and its effect on target genes. Mouse macrophages isolated from MAS mice liver were transfected with miRNA mimics, inhibitors, or respective negative controls. Relative expression of target/s of each miRNA mimic or inhibitor was calculated against the respective negative control following qRT-PCR analysis. Relative expression of targets of **(A)** miR-136 and miR-501 involved Fcγ receptor–mediated phagocytosis and **(B)** miR-129-1and miR-150 involved in Il-12 production. Vertical bars represent mean ± SEM. Mimic and inhibitor were compared for statistical significance using Student’s t-test. *p < 0.05, **p < 0.01, ***p < 0.005 and ****p < 0.0001.

We performed miRNA transfection experiments to confirm the link between specific miRNA and their targets. To that end, macrophages were isolated from MAS mice and transfected with mimics and inhibitors of miRNAs. The data demonstrated that the mimic of miR-136 decreased the expression of Fcgr4, whereas the inhibitor of miR-136 increased the expression of Fcgr4 (**Figure 5A**). The mimic decreased the expression of Fcgr3, whereas the inhibitor did not have a significant effect on Fcrg3. In addition, the mimic of miR-501 decreased the expression of Fcgr1, whereas the inhibitor of miR-501 increased the expression of Fcgr1 (**Figure 5A**). Experiments using mimics and inhibitors of miR-129-1 and miR-150 suggested that mimics did not have significant impact on mRNA expression, whereas inhibitors increased the expressions of Il12b and Il12a, respectively (**Figure 5B**).

### 3.6 miR-136 and miR-501 target expression of genes in phagocytosis pathways other than FGRP

Following target analysis of miRNA data set against the RNA gene expression profile using IPA and miRWalk (28), we found that both miR-136 and miR-501 target the expression of multiple genes involved in PRRs, FCϵRI, and phagosome formation. **Table 1** shows the miRNA targets in above phagocytosis-associated pathways.

**Table 1:** Targets of miR-136 and miR-501 in pattern recognition receptors (PPRs), Fc epsilon RI and phagosome formation.

Gene	Blood (GSE57253)	PBMNCs (GSE38849)	Monocytes (GSM4435366)	Monocytes (GSM4435367)
*FCGR1A*	3.07	4.51	46.84	29.87
*FCGR1B*		5.41	4.33	4.04
*FCGR2A*	1.54		3.47	1.92
*FCGR3A*	2.01	2.37	16.22	11.3
*FCGR3B*	2.28	13.23	53.79	8.2
*FCGRT*	1.98		4.33	4.4
*FCGR2B*	1.28	-2.8	3.71	2.01
*CD163*	3.02		2.48	3.1

### 3.7 miRNAs regulate phagocytosis in macrophages

**Figure 6:**
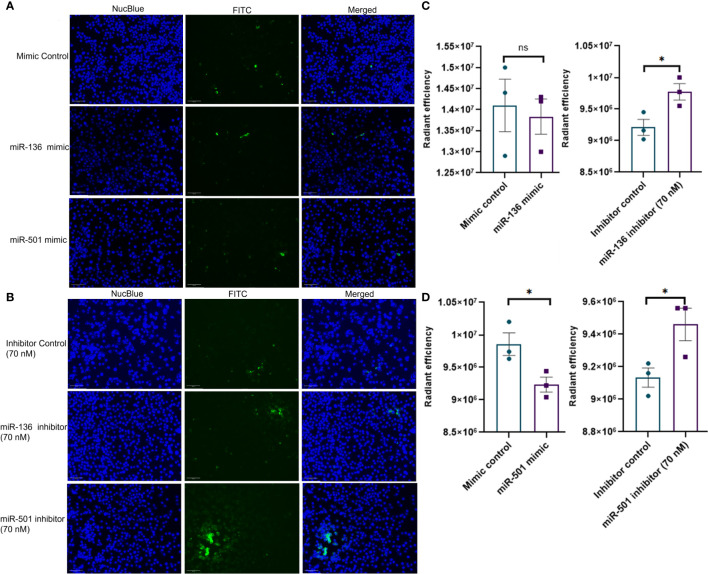
Modulation of IgG-mediated phagocytosis by miR-136 and miR-501. RAW cell line was transfected with miRNA mimics, inhibitors, or the respective controls, and phagocytosis assay was performed using FITC-conjugated IgG. Fluorescence was quantified at excitation/emission of 465/540 and represented as radiant efficiency. Live cells were imaged following the addition of IgG and staining with NucBlue. Fluorescent microscopy images of cells stained with NucBlue and FITC and merged following transfection with **(A)** mimics and **(B)** inhibitors (20×; scale bar, 100 µm). Radiant efficiency for **(C)** miR-136 mimic and inhibitor and **(D)** miR-501 mimic and inhibitor. Vertical bars represent mean ± SEM. Mimic/inhibitor control and treated as well as MAS and control mice were compared for statistical significance using Student’s t-test. *p < 0.05.

Next, we investigated if altered expression of miR-136 and miR-501 would directly affect the phagocytic functions of macrophages. To that end, we used two phagocytosis assays. In one assay, we used FITC-conjugated IgG particles to assess the ability of miR-136 and miR-501 to modulate FGRP. The fluorescent microscopy images showed phagocytosis as green fluorescence inside macrophages following the engulfment of IgG particles (**Figures 6A, B**). Quantification of fluorescence showed a significant decrease in phagocytosis following transfection with mimic of miR-501 although miR-136 did not decrease phagocytosis significantly (**Figures 6C, D**). A significant increase in phagocytosis with 70 nM concentration of inhibitors of miR-136 and miR-501 was also observed (**Figures 6C, D**).

**Figure 7:**
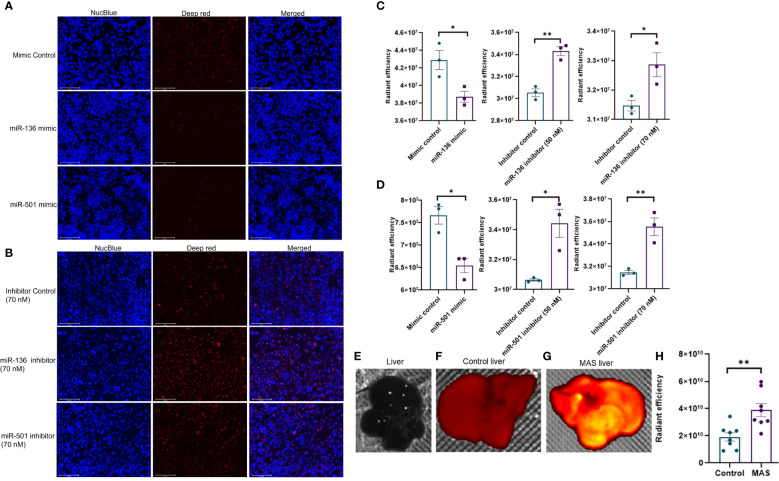
*In vivo* phagocytosis in mice with MAS and its regulation by miRNA *in vitro*. RAW cell line was transfected with miRNA mimics, inhibitors, or the respective controls, and phagocytosis assay was performed using pHrodo deep red *E coli* bioparticles. Fluorescence was quantified at 640/680 excitation/emission and represented as radiant efficiency. Live cells were imaged following the addition of bioparticles and staining with NucBlue. Fluorescent microscopy images of cells stained with NucBlue and deep red and merged following transfection with **(A)** mimics and **(B)** inhibitors (20×; scale bar, 100 µm). Radiant efficiency for **(C)** miR-136 mimic and inhibitor and **(D)** miR-501 mimic and inhibitor. Control and MAS mice were injected with pHrodo deep red *E coli* bioparticles intravenously, and fluorescence was imaged and quantified using IVIS Spectrum *in vivo* imaging system. Respective images of **(E)** normal liver without particles injected, liver from **(F)** control and **(G)** MAS mouse following bioparticles injection, and **(H)** quantification of fluorescence. Vertical bars represent mean ± SEM. Mimic/inhibitor control and treated as well as MAS and control mice were compared for statistical significance using Student’s t-test. *p < 0.05 and **p < 0.01.

The second phagocytosis assay involving deep red *E. coli* bioparticles made use of the pH change inside phagosomes for the detection of phagocytosis. The red fluorescence becomes visible only when pH becomes acidic inside phagosomes, whereas the medium outside phagosomes remain pH neutral. As a result, an increase in fluorescence indicates an increase in phagocytosis. The fluorescent microscopy images showed phagocytosis as red fluorescence inside macrophages following the engulfment of bioparticles (**Figures 7A, B**). Quantification of fluorescence showed a significant decrease in phagocytosis following transfection with mimics of both miR-136 and miR-501 (**Figures 7C, D**). An increase in phagocytosis with both 50 and 70 nM concentrations of miRNA inhibitors of miR-136 and miR-501 was also observed (**Figures 7C, D**). Next, we tested if mice with MAS exhibit increased phagocytosis. To that end, we performed an *in vivo* phagocytosis assay following a previously published method (22). The intravenous injection of fluorescent-labeled *E. coli* particles significantly increased the total fluorescence of liver from MAS mice compared to control (**Figures 7E–H**), thereby suggesting an overall increase in phagocytosis in MAS liver when compared to the controls.

### 3.8 FGRP is upregulated in patients with MAS

**Figure 8:**
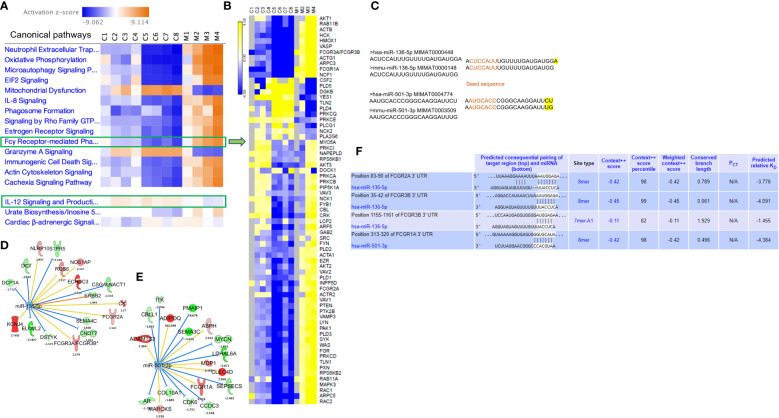
Gene expression profiling and pathway enrichment analysis of normal control (C) and patients with MAS (M). **(A)** Canonical pathways following comparison of gene expression profiles and pathway enrichment analysis of normal controls and patients with MAS. **(B)** Heat map demonstrating fold change expression of genes in controls and patients with MAS in FGRP. **(C)** Sequence similarity of human and mouse miR-136-5p and miR-501-3p. **(D)** Selected targets of miR-136-5p and **(E)** miR-501-3p in patient with MAS. **(F)** TargetScan analysis of miR-136 and miR-501 binding to the respective 3′ UTR of target genes.

Owing to the increased expression of mouse phagocytic receptors Fcgr1 (CD64), Fcgr3 (CD16), and Fcgr4 (CD16-2) in murine MAS, we investigated these markers in patients with MAS. To achieve this, we retrieved raw data from whole RNA sequencing of three MAS patient samples as well as CEL file of microarray analysis of one patient with MAS that were available in the GEO (NCBI). The RNA sequencing data were analyzed using CLC-Genomics Workbench and microarray data by Transcriptome Analysis Console software. Details of MAS patient and control data sets used are provided in **Supplementary Table 1**. A comparison between four patients with MAS and eight normal controls identified FGRP as one of the most significant pathways upregulated in patients with MAS with positive Z scores ranging from 4 to 7 (**Figure 8A**). Gene expression profile derived from the microarray data did not produce a significant Z score possibly because the analysis identified only 12 genes involved in FGRP, whereas pathway analysis of other MAS patients’ data sets identified more than 70 genes involved in FGRP (**Figure 8B**). It was exciting to note that there was a substantial increase in the expression of human orthologs of mouse phagocytic genes in all four patients with MAS. *FCGR1A*− human ortholog of murine Fcgr1, *FCGR2A* (CD32), and *FCGR3A/3B*− human orthologs of murine Fcgr3 and Fcgr4, respectively, were overexpressed in patients with MAS along with other FGRP promoting genes. Of note, the gene expression profile from the microarray data analysis also showed an increased expression of FCGR genes signifying the potential role of these genes in MAS pathogenesis. A heat map (generated by Morpheus, https://software.broadinstitute.org/morpheus) illustrating fold change expression of genes participating in FGRP is provided in **Figure 8B**. Of the four patient samples, two were data from peripheral blood and two were from monocytes. As expected, the fold change expression of monocyte FGRP genes was greater than the peripheral blood FGRP genes. Fold change expression of the macrophage hemophagocytic marker *CD163* was smaller on monocytes than that of FCGR genes. **Table 2** depicts the fold change expression of FCGR genes and *CD163* from each patient with MAS. The consistent pattern of overexpression of FGRP correlated with the increased phagocytosis in MAS mice and hemophagocytosis in patients with MAS. IL-12 production pathway was not significantly altered in patients with MAS (**Figure 8A**).

**Table 2:** Fold change expression of Fcγ receptor genes and *CD163* in MAS patients.

miRNA	mRNA targets in phagocytosis-associated pathways Fold change in parentheses		
miR-136	PRR	Fc Epsilon RI	Phagosome formation
	Ifnγ (5.43) Tnfsf14 (1.44) Tnfsf15 (5.22) Tnfsf8 (−2.15) Nfkbie (1.46)		Fgr (1.6) Gipr (−3.23) Gpr141 (3.55) Itgb5 (1.7) Msr1 (2.89) Prokr1 (1.86) Tuba3b (−4.21)
miR-501	Ikbkb (1.53) Ikbke (4.7) Tlr1 (4.41) Tlr9 (1.54) Tlr7 (2.86)	Mapk14 (1.47) Rap1a (1.25)	Adgra2 (−1.75) Adora3 (4.16) Atp6v1a (2.18) Ccr5 (2.94) Ctsa (1.98) Itga2b (2.96) Oprk1 (−7.16) Pip4k2a (1.91) Tlr12 (1.78)

### 3.9 miR-136 and miR-501 target FCGR gene expression in patients with MAS

**Figure 9:**
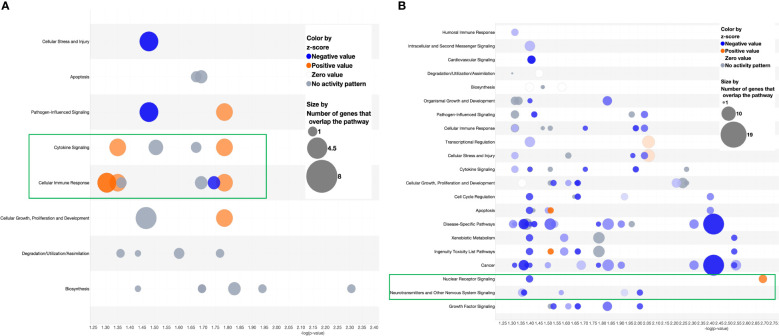
Bubble chart derived from the target analysis of **(A)** miR-136-5p and **(B)** miR-501-3p against a MAS patient gene expression data.

Following the discovery of a robust increase in FCGR gene expression in patients with MAS, we next explored whether the miRNAs controlling phagocytosis in mice are structurally similar to their counterparts in humans. To that end, we compared the miRNA sequences of mmu-miR-136-5p and hsa-miR-136-5p as well as mmu-miR-501-3p and hsa-miR-501-3p from miRBase (29). Surprisingly, the comparison revealed that all the nucleotides of mmu-miR-136-5p were shared with hsa-miR-136-5p with the latter having one additional nucleotide added to the 3′ end. At the same time, there was a difference of two nucleotides between mmu-miR-501-3p and hsa-miR-501-3p at the 3′ end, but the seed sequences remained the same (**Figure 8C**). This suggested that the miRNAs might bind to the same targets in humans as well. To explore this further, we made use of the miRNA target filter tool of IPA. The miRNA target analysis of IPA uses a prediction method for the confidence of a match between a miRNA with a specific seed sequence and an mRNA. Because the seed sequences of miRNAs under study are the same between mice and humans, we concatenated the mouse miRNA data set with the differential gene expression data derived from the peripheral blood of a patient with MAS (GSE57253). As anticipated, the analysis identified *FCGR2A* and *FCGR3A/3B* as targets of miR-136-5p and *FCGR1A* as the target of miR-501-3p (**Figures 8D, E**). We used TargetScanHuman 8.0 (30) to confirm the results and ascertained that the hsa-miR-136-5p binding sequence is indeed present in the 3′ untranslated region (UTR) of *FCGR2A* and *FCGGR3B* and hsa-miR-501-3p binding sequence in the 3′ UTR of *FCGR1A* (**Figure 8F**). Because miRNAs target several genes, it is essential to know the genes and pathways that the miRNAs can regulate. To that end, a network of miR-136-5p target genes was created from the same MAS patient data. The following parameters were set to identify the targets; a fold change difference of > or < 1.5 and highly predicted genes with a known biological function (**Figures 8D, E**). For the pathway enrichment analysis, we selected moderate and highly predicted gene targets with a fold change expression of < or > 1.2. The miR-136 target genes pathway analysis revealed upregulation of pathways involved in cytokine signaling and immune response in patients with MAS (**Figure 9A**). The targets of miR-501 were involved in reduced xenobiotic metabolism, neurotransmitters, and other nervous system signaling and cancer (**Figure 9B**).

## 4 Discussion

MAS is a life-threatening complication of sJIA that primarily affects children. Although it can occur in any rheumatic disease, it is more commonly associated with sJIA with ~10% of the patients with sJIA developing MAS. It is an under-investigated hyper-inflammatory condition, culminating in cytokine storm, fever, rashes, peripheral pancytopenia, hyperferritinemia, hepatosplenomegaly, elevations in C-reactive protein concentration, and hemophagocytosis (1). Decreasing platelet and WBC counts are proposed as indicators of MAS in sJIA patients with sJIA from sJIA flares (31). Diagnosis of MAS when associated with other underlying inflammatory or autoimmune diseases remains a challenge. Clinical features of MAS overlap with the guidelines for identification of HLH and sJIA, whereas the classification criteria do not satisfactorily differentiate patients with MAS from those with HLH and sJIA (4). Thus, more research is needed for the specific and early diagnosis of MAS. MAS is also characterized by cytokine storm and hemophagocytosis, and the mechanism of pathogenesis remains unclear.

In the current study, we investigated the role of miRNA in the activation of macrophages in a well-established murine model of MAS, in which MAS was induced by repeated stimulation of TLR-9 by the oligonucleotide, CpG. This experimental model has been reported to induce pathological manifestations of MAS in murine models. Clinical characters of MAS such as cytopenia, cytokine storm, hepato-splenomegaly, and hyperferritinemia were replicated in this murine model of MAS as reported by others (8, 11). Hemophagocytosis is reported in this model of MAS where splenic hemophagocytosis was observed following blocking of IL-10 and the role of IFN-γ to mediate anemia was shown to be not necessary for fulminant TLR-9–induced MAS and hemophagocytosis (32).

Our pre-clinical model of MAS demonstrated cytopenia, cytokine storm, hepatosplenomegaly, and hyperferritinemia. The increase in Ly6C and F4/80-positive cell populations demonstrated the expansion of monocyte and macrophage populations in MAS mice. We also observed a significant increase in CD11B and CD11C double-positive cells that also indicated expansion of monocytes/macrophages/DCs. Along with macrophages and monocytes, elevated DCs are reported in mouse models of MAS where depletion of DCs partially reduced circulating IFN-γ (14, 24). Although initially reported as IFN-γ–dependent, later studies established IFN-γ–independent establishment of MAS characteristics in murine models (32, 33).

Liver tissues of patients with MAS have been reported to demonstrate increased number of macrophages, and hemophagocytosis was detected in the cytoplasm of macrophages following immunohistochemical staining, whereas bone marrow aspirates did not always show hemophagocytosis (2). Hemophagocytosis is the phagocytosis of erythrocytes, leukocytes, platelets, and other hematopoietic precursors by cells of monocyte/macrophage or DC lineage and is identified as a mechanism behind cytopenia (34–36). The reason behind the destruction of blood cells in hemophagocytic syndromes has been largely attributed to macrophages (23, 37). Although proved in patients with MAS, phagocytosis was not given much attention when studying pathogenesis of MAS in preclinical models and controlling phagocytosis has not been explored as a therapeutic strategy. Macrophage receptor CD163 is the established marker to detect hemophagocytosis in patients with MAS (23, 38). However, we did not find an increase in the expression of Cd163 (−1.5-fold change) in the liver of MAS-bearing mice. This observation was consistent with a previous report of negative Cd163 immunohistochemical staining for spleen and bone marrow in a mouse model of MAS (14). In humans, CD163 takes part in the scavenging of hemoglobin by promoting the uptake of haptoglobin-hemoglobin complex, leading to red blood degradation. Contrary to human hemoglobin, mouse hemoglobin did not display elevated affinity binding to CD163 (39). CD163 is mainly expressed in M2 macrophages in the human spleen. A previous report showed that serum soluble CD163 levels were elevated in patients with s-JIA (40). These findings indicated that the activation marker of CD163 can be transformed into a soluble form in the blood, and soluble CD163 can be associated with the activation of M2 macrophages in the spleen of patients with s-JIA. Therefore, the low levels of CD163 gene expression in peripheral blood may not always indicate the absence of M2 macrophage activation. We demonstrated increased phagocytosis in the liver of MAS mice, which aligned with cytopenia detected in the peripheral blood of these mice. Phagocytosis is mediated by opsonic and non-opsonic mechanisms. Fc receptors such as FCGRs (Fc*γ*RI, Fc*γ*RII, and Fc*γ*RIII) and FCϵRI as well as complement receptors are opsonic receptors. Various isoforms of FCGRs are associated with phagocytosis by macrophages and DCs (41, 42). FCGR genes belong to the immunoglobulin superfamily and play a crucial role in phagocytosis of opsonized microbes and immune complexes, and blocking or neutralizing Fc receptors has been proposed as a therapeutic approach to alleviate chronic inflammation and autoimmune disorders (43–46). PPRs such as Toll-like receptors are the non-opsonic receptors mediating phagocytosis of non-opsonized particles (47). In the current study, using RNA sequencing and qRT-PCR, we found elevated expression of genes involved in FGRP in macrophages, specifically Fcgr1, Fcgr3, and Fcgr4. FCGR genes are reported to promote macrophage phagocytosis and platelet clearance in patients with immune thrombocytopenia and blocking FC receptors demonstrated to be an effective treatment strategy (45, 46, 48, 49). No significant activation of complement system was observed following pathway enrichment analysis of MAS mice or human gene expression data. Interestingly, bulk RNA sequencing of MAS spleen cells showed downregulation phagocytosis-associated pathways (data not shown). Previous studies described that hemophagocytosis is not induced in the spleen of MAS mice and blocking of IL-10 was required along with CpG administration for induction hemophagocytosis in the spleen (14, 32).

There are no investigations so far on the epigenetic modulation of MAS, and the current study presents miR-136-5p and miR-501-3p as important miRNAs modulating the pathogenesis of MAS. In our study, we identified miRNAs targeting macrophage/monocyte mediated phagocytosis through the regulation of FCGR. Specifically, we identified miR-136-5p and miR-501-3p to augment FCGR gene expression. We demonstrated regulation of the miRNA targets in macrophages of MAS-bearing mouse using synthetic mimics and inhibitors of miRNAs. In addition, the ability of both miR-136 and miR-501 to target gene expression and modulate phagocytosis via pathways other than FGRP was confirmed by the deep red *E. coli* phagocytosis assay. The role of miR-136-5p in the regulation of macrophage functions has not been previously reported. Primarily, miR-136-5p has been shown to act as a tumor-inhibitor in a variety of cancer models (50). Similarly, whereas the function of miR-501-3p that we found to target Fcgr1 has not been characterized in macrophages, it was found to act as tumor suppressor in non-small cell lung cancer (51). In addition, miR-501-3p has previously been identified as a possible biomarker related to disease progression in patients with Alzheimer's disease where its expression was deregulated (52). Functional enrichment analysis of the significantly altered miRNAs in MAS mouse revealed their importance in central nervous system disorders, a clinical feature reported in patients with MAS.

IL-12 is primarily produced by DCs, macrophages, and neutrophils (53). IL-12 is well established for T-cell activation and induction of other inflammatory cytokines such as IFN-γ and TNF-α (54, 55) consistent with the observation that such cytokines were upregulated during MAS (14). An elevated level of IL-12 was detected in the serum of MAS mice and patients with HLH (56, 57).

We also found that miR-129-1-3p and miR-150-3p expression was downregulated in MAS and that these miRNAs targeted Il12b and Il12a gene expression, respectively. miR-129-3p was reported to act as a tumor repressor and a promoter of cancer resistance (58, 59). miR-129-3p has also been shown to inhibit the expression of inflammatory cytokine IL-17 in rheumatoid arthritis (60). Increased expression of miR-150-3p was also related to pulmonary improvements in patients with COVID-19 with lung injury (61). Overall, these data suggested that overexpression of miR-150-3p may be associated with anti-inflammatory effects by suppressing inflammatory cytokines such as IL-12 or IL-17. Controlling cytokine storm is the most pursued treatment strategy to subdue MAS with steroids and cytokine-directed therapies being the first line of treatment choices. Existing treatment modalities exploit inhibitors of inflammatory cytokines such as IL-1β and IL-6. Anakinra, an IL-1β blocker, and tocilizumab, an IL-6 blocker had been used in clinics to treat MAS albeit with side effects and limited efficacy (56, 62). An *NLRC4* mutation in patients with MAS was reported to increase the production of IL-18, and neutralization of IFN-γ reversed the clinical and laboratory characteristics of MAS in a mouse model (8, 10, 56), expanding the possibilities of uncovering more cytokine blockers.

In the current study, we did not investigate the mechanism through which miRNAs regulate the gene expression, which is a limitation. Binding of miRNA to the 3′ UTR of target gene is one of the mechanisms of miRNA-mediated gene silencing (63). The binding of miRNA to the coding sequence of target genes (64–66) and 5′ UTR (67, 68) are the other well-established mechanisms that cause mRNA silencing.

In the current study, we tried to correlate our findings using mouse model of MAS with MAS patient data from four patients. To the best of our knowledge, only four MAS patient data are available for accession in a public domain. Transcriptome analysis of MAS patient data showed FGRP as one of the most significant pathways upregulated in patients with MAS. We found an increase in the expression of human orthologs of mouse phagocytic genes in all four patients with MAS. *FCGR1A*− human orthologs of murine Fcgr1, *FCGR2A*, and *FCGR3A/3B*− human orthologs of murine Fcgr3 and Fcgr4, respectively, were overexpressed in patients with MAS along with other FGRP promoting genes.

miRNA target identification based on the binding of miR-136-5p and miR-501-3p seed sequence to the respective target genes confirmed their role in regulating FCGR gene expression in patients with MAS, when MAS patient gene expression data were concatenated for target analysis. CD163, identified in the bone marrow and liver biopsies of patients, has been considered as a diagnostic marker in MAS (23, 38). However, the gene expression profile from MAS patient monocytes from the current study showed diminished expression of *CD163* compared to *FCGR* genes (**Table 2**). Based on the overexpression of *FCGR* genes in humans and the mouse model of MAS along with the identification of FGRP as a major pathway upregulated in patients during MAS, we propose that *FCGR* gene expression may be considered as a biomarker of MAS. This is also supported by the demonstration of increased *in vivo* phagocytosis in the liver of MAS-bearing mice. Nonetheless, this has to be confirmed by comparing the gene expression profiles of patients with MAS against the gene expression data sets of patients with sJIIA and HLH.

miRNA-targeted therapeutics are under clinical development to treat various disorders (69). An example is the clinical development of TargomiRs that are mimics of miRNAs transported and delivered by targeted microbial minicells (70). Development of TargomiR loaded with miRNA-16–based mimic launched the first clinical trial (NCT02369198) of a miRNA mimic to treat patients with recurrent malignant pleural mesolithoma (71). A recent gene therapy modality uses AMT-130 that contains a miRNA to bind and stop the translation of huntingtin mRNA to treat Huntington’s disease (NCT04120493). Individual or combined blockade of *FCGR1* and *FCGR3* is proposed to be an effective therapeutic strategy to treat immune thrombocytopenia (49). We established in the current study that upregulating miR-136-5p and miR-501-3p can downregulate the expression of these receptors. Thus, the use of such miRNA mimics may help block the induction of target genes involved in the pathogenesis of MAS in humans.

## Data availability statement

The data presented in the study are deposited in the National Center for Biotechnology repository, accession number PRJNA1082738.

## Ethics statement

The animal study was approved by Protocol AUP-2669-101803, University of South Carolina Institutional Animal Care and Use Committee. The study was conducted in accordance with the local legislation and institutional requirements.

## Author contributions

KV: Writing – review & editing, Writing – original draft, Visualization, Validation, Software, Methodology, Investigation, Formal analysis, Data curation. XY: Writing – original draft, Methodology. AC: Writing – original draft, Methodology. YZ: Writing – original draft, Methodology. MN: Writing – review & editing, Supervision, Resources, Project administration, Funding acquisition, Conceptualization. PN: Writing – review & editing, Supervision, Resources, Project administration, Funding acquisition, Conceptualization.
