# Supplementary material for: Identification of miRNAs that target Fcγ receptor-mediated phagocytosis during macrophage activation syndrome
Source: Front Immunol. 2024 Mar 15;15:1355315. doi: 10.3389/fimmu.2024.1355315 (PMC10981272; doi:10.3389/fimmu.2024.1355315)
Supplement: Supplementary file 1 [file DataSheet_1.pdf]

Supplemental Table 1. GEO accession number and sample IDs of MAS patient data sets

**GEO data set**	**Sample ID**	**Disease/Control**	**Sample type**	**Ferritin level** (ng/mL)
GSE57253	GSM1377977<br>GSM1377978<br>GSM1377979	Control	Peripheral blood	
	GSM1377956<br>GSM1377957<br>GSM1377958	MAS (sample from same patient at 3 time point)	Peripheral blood	5605
GSE147608	GSM4435366<br>GSM4435367	MAS	Monocytes	8209<br>16464
	GSM4435388<br>GSM4435381<br>GSM4435385<br>GSM4435391	Control	Monocytes	
GSE38849	GSM340372	MAS	PBMNCs	2416
GSE7753	GSM187587	Control	PBMNCs	

Supplemental Table 2. Primers used for miRNA validation.

miRNA	Primer
miR-136-5p	5'ACUCCAUUUGUUUUGAUGAUGG
miR-501-3p	5'AAUGCACCCGGGCAAGGAUUUG
miR-129-1-3p	5'AAGCCCUUACCCCAAAAAGUAU
miR-150-3p	5'CUGGUACAGGCCUGGGGGAUAG

Supplemental table 3. Primers used for target genes validation.

Primer	Forward	Reverse
Fcgr1	aac agc cgt tca gat ctc ca	ttc cat ccg tga cac ctc aa
Fcgr3	cca gct aca cgt tta agg cc	tag cgt gat ggt ttc ccc tt
Fcgr4	gtg gct cct act tct gca ga	ttg tcc tga ggt tcc ttg ct
Il12a	cat cga tga gct gat gca gt	cag ata gcc catcac cct gt
Il12b	gac atg tgg aat ggc gtc tc	tta ttc tgc tgc cgt gct tc
18s	ctc aac acg gga aac ctc ac	cgc tcc acc aac taa gaa cg
Gapdh	atg gtg aag gtc ggt gtg aa	atg tta gtg ggg tct cgc tc

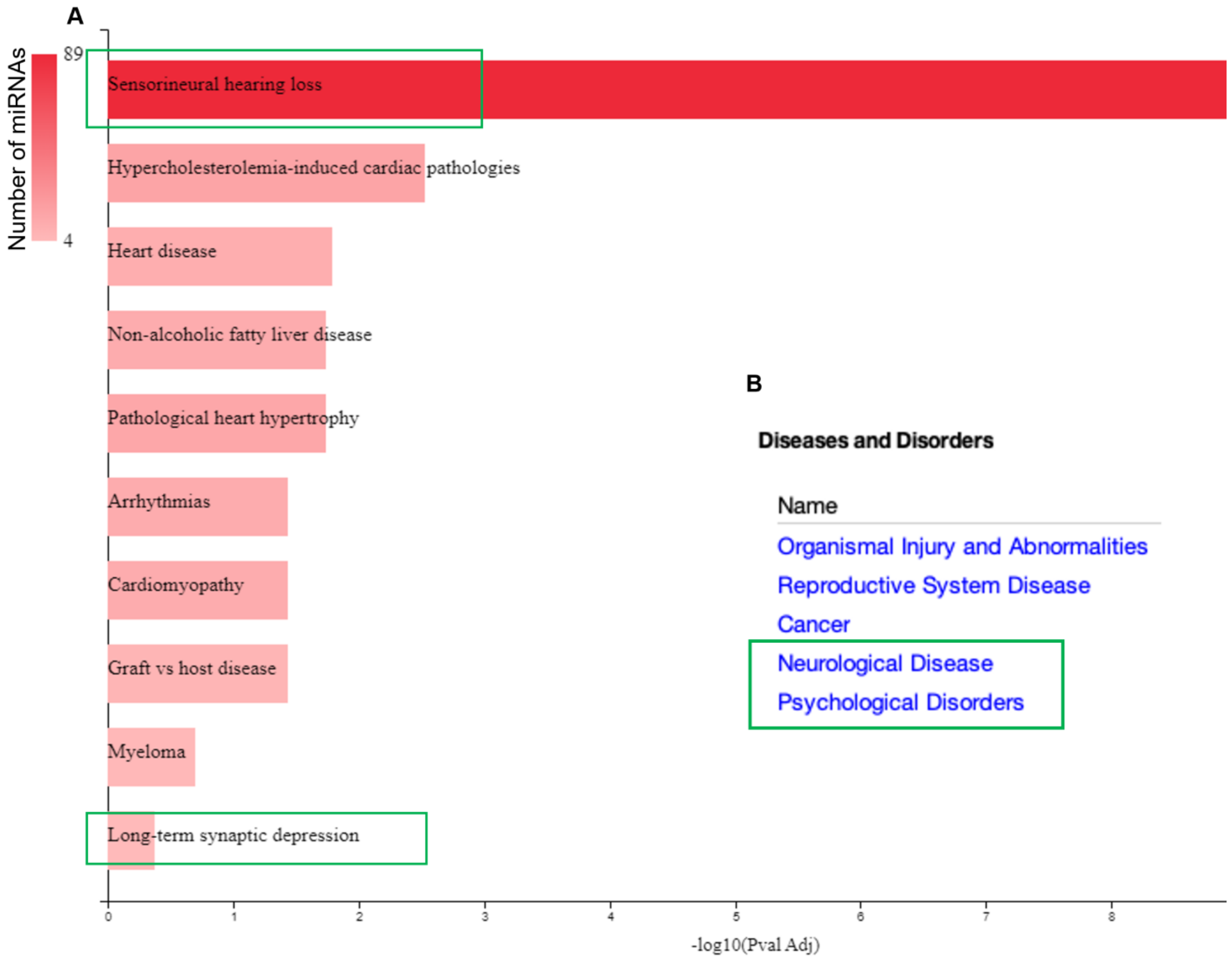

Supplemental Figure 1: Diseases associated with the significantly altered miRNAs in MAS mice. (**A**) GeneCodis and (**B**) IPA generated diseases/disorders related to miRNA expression in MAS.

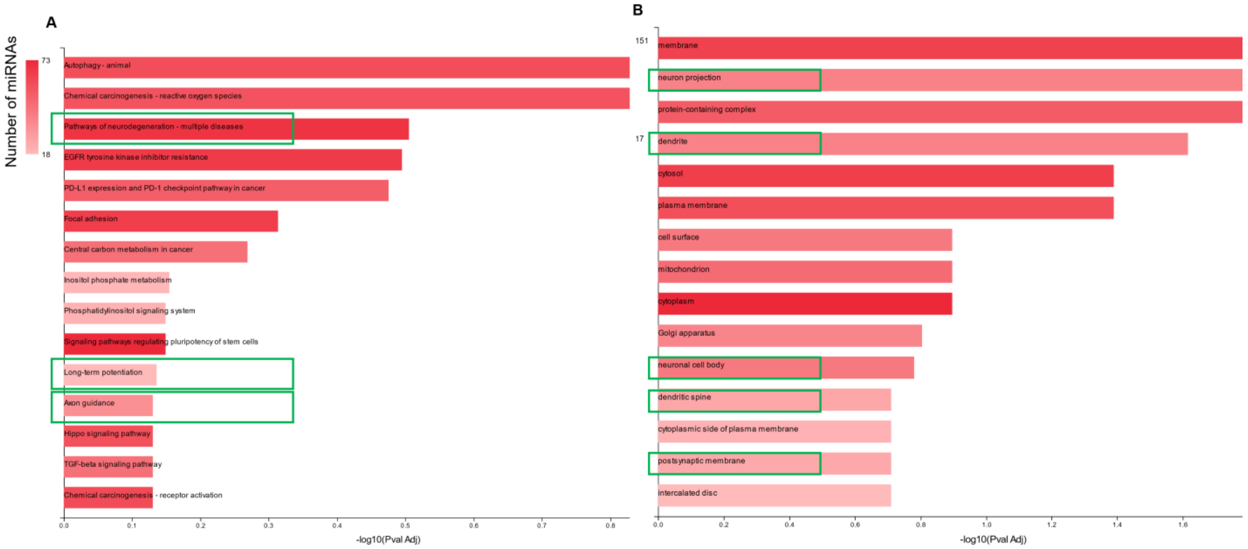

Supplemental Figure 2: Pathway and cellular component analysis of miRNAs in MAS mice generated by GeneCodis. (**A**) KEGG pathway and (**B**) cellular component analysis of significantly altered miRNAS.
